# Trajectories of cardiovascular ageing—from molecular mechanisms to clinical implementation

**DOI:** 10.1093/cvr/cvae178

**Published:** 2024-08-23

**Authors:** Stefano Ministrini, Florian A Wenzl, Thomas F Lüscher, Giovanni G Camici

**Affiliations:** Center for Molecular Cardiology, University of Zurich, Wagistrasse 12, 8952 Schlieren, Switzerland; Center for Molecular Cardiology, University of Zurich, Wagistrasse 12, 8952 Schlieren, Switzerland; Disease Registration and Analysis Service, National Health Service, London, UK; Department of Cardiovascular Sciences, University of Leicester, Leicester, UK; Department of Clinical Sciences, Karolinska Institutet, Stockholm, Sweden; Center for Molecular Cardiology, University of Zurich, Wagistrasse 12, 8952 Schlieren, Switzerland; Royal Brompton and Harefield Hospitals, Imperial College and King’s College, London, UK; Center for Molecular Cardiology, University of Zurich, Wagistrasse 12, 8952 Schlieren, Switzerland; Department of Research and Education, University Hospital Zurich, Zurich, Switzerland

**Keywords:** Cardiovascular ageing, Ageing hallmarks, Epigenetic clocks, Sirtuins, Autophagy, Inflamm-ageing

## Abstract

Due to its peculiar structure and function, the cardiovascular system is particularly vulnerable to the detrimental effects of ageing. Current knowledge about the molecular mechanisms of ageing revealed the processes actively promoting ageing, e.g. progressive telomere shortening, and the mechanisms opposing it, e.g. endogenous production of antioxidant substances. This knowledge can be used to measure biological age at cellular and molecular levels and to interfere with it by pharmacological or non-pharmacological interventions. Biological ageing is determined by the simultaneous occurrence of independent hallmarks, which encompass a wide range of biological processes, from genomic changes to systemic inflammation and dysbiosis. This narrative review will summarize the role of ageing hallmarks in the cardiovascular system, how they can be measured, and what are the possible interventions to counteract their effects.

## Graphical Abstract

**Graphical Abstract:**
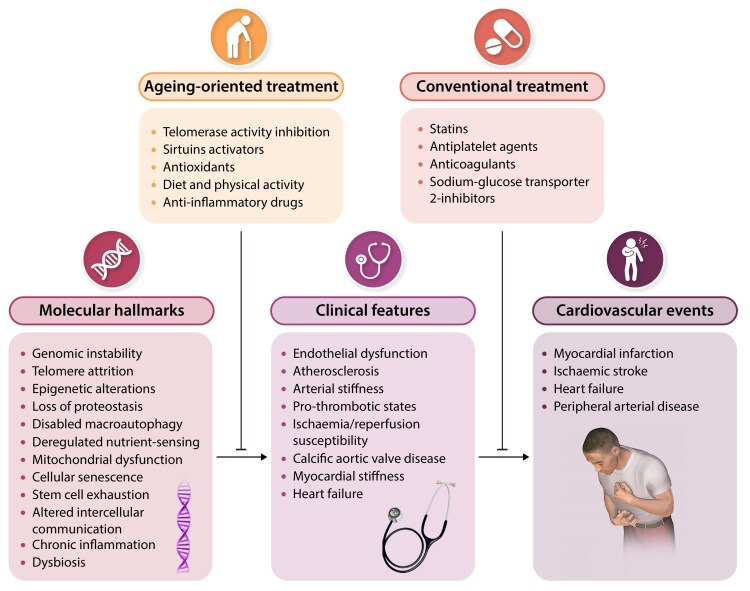
Ageing-oriented strategies for prevention and treatment of cardio- and cerebrovascular diseases. Compared to conventional strategies for prevention and treatment of CVD and CBVD, which aim at controlling the progression of the risk factors to overt disease or the progression of the disease towards disability and death, ageing-oriented treatments aim at slowing down the progression of molecular and cellular ageing towards the overt risk factor.

**This article is part of the Spotlight Issue on Ageing.**

## 1. Introduction

**Figure 1:**
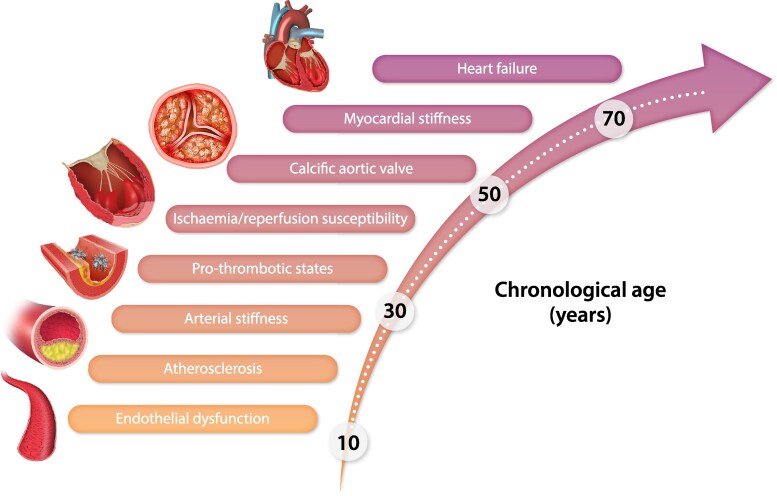
Trajectories of cardiovascular ageing. Ageing in the cardiovascular system is characterized by the occurrence of eight main pathological features. Ageing-related cellular and molecular changes start very early in adult life and then they slowly progress to constitute the overt pathological features with different progression rates. The figure represents the progressive occurrence of the ageing-related pathological features with the typical average age of occurrence.

Cardiovascular and cerebrovascular diseases (CVD and CBVD) are the leading causes of death and disability worldwide,^1^ and their high incidence is an undesired consequence of the generalized increase in human life expectancy.^2^ Because of its peculiar structure and function, the cardiovascular system is particularly vulnerable to the detrimental effects of ageing; hence, age is the main risk factor for CVD and CBVD. Ageing of the cardiovascular system is characterized by the occurrence of eight main pathophysiological features, summarized in *Figure 1*. They appear very early in life (beginning from 25 years of age) at the molecular level, and then, they slowly progress to constitute the established cardiovascular and cerebrovascular risk factors, eventually leading to CBVD and CVD.

Historically, we are still used to considering age as a non-modifiable risk factor, strictly dependent on its numeric value; however, advances in ageing biology revealed that biological age does not necessarily correspond to chronological age. This led to explore the possibility of measuring biological age at cellular and molecular levels,^3^ as well as modifying the process of biological ageing by pharmacological or lifestyle interventions, in order to interfere with its progression.^4^ Compared to conventional strategies for prevention and treatment of CVD and CBVD, which aim at preventing the progression of risk factors to overt disease or the progression of the disease towards disability and death, ageing-oriented treatments strategies aim at blunting the progression of molecular and cellular ageing towards the overt risk factor (*Graphical Abstract*). Under this perspective, ageing-oriented treatments represent a modern frontier in precision cardiology.

In this narrative review, we will summarize the molecular mechanisms of cardiovascular ageing and highlight the potential interventions to counteract their effects in a potential, future clinical setting. Given the increasing interest paid to this topic, several papers reviewed the recent advances in the field of cardiovascular ageing.^5–8^ In this review, we aim at offering a general overview of the molecular mechanisms underlying cardiovascular ageing, according to the most recent theoretical framework on ageing, as described by Lόpez-Ótin *et al.*^9^ Furthermore, we aim at building a bridge between this framework and potential clinical applications, by describing potential ageing-oriented interventions in a hallmark-specific manner.

## 2. Molecular mechanisms of cardiovascular ageing

Ageing was historically considered a passive process, resulting from the exhaustion of homeostatic mechanisms and the subsequent accumulation of cellular and molecular damage over time. Conversely, current knowledge about the molecular mechanisms of ageing reveals the existence of pathways opposing ageing, as well as biochemical processes actively promoting its progression.

**Figure 2:**
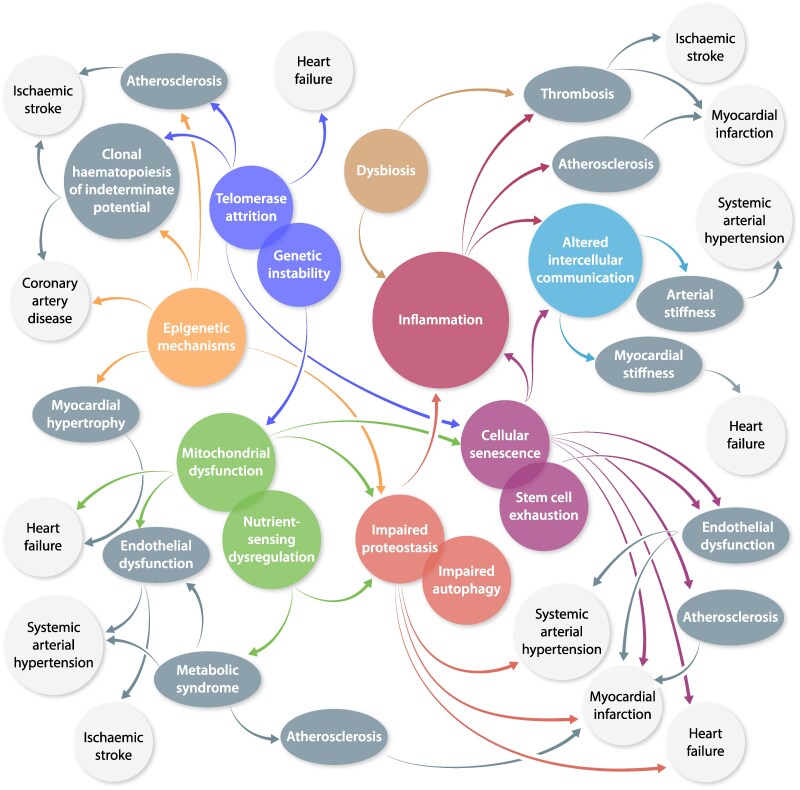
Hallmarks of ageing in cardiovascular (CV) of ageing: an expanding galaxy. Hallmarks are depicted by coloured balloons. Tightly connected hallmarks have the same colour and are overlapped. Coloured arrows connect the hallmarks to each other and to pathogenetic CV factors (grey ovals). Grey arrows connect pathogenetic CV factors to overt disorders (white squares).

According to Lόpez-Ótin *et al.*,^9^ biological ageing is determined by the co-occurrence of 12 independent hallmarks, which encompass a wide range of biological processes, from molecular changes to systemic dysregulation. The effect of each hallmark in the pathogenesis of age-related CVD and CBVD is summarized in *Figure 2*.

The final consequence of ageing is frailty, defined as the concurrent exhaustion of multiple physiological compensatory mechanisms, thus leading to an increased vulnerability to stressors. The relationship between frailty and CVD is particularly complex and involves multiple different factors, including psycho-social and economic conditions. In this review, we mainly focus on the molecular mechanisms of cardiovascular ageing and the possibility to counteract these mechanisms with targeted interventions. The complex relationship between frailty and CVD/CBVD has been extensively reviewed elsewhere.^10^

### 2.1 Ageing genome: genomic instability and telomere attrition

Integrity and stability of genetic information are fundamental prerequisites for cellular function. The role of genomic instability in cardiovascular ageing, however, is still controversial. Subjects with hereditary conditions characterized by increased genomic instability, such as Hutchinson–Gilford progeria syndrome or ataxia telangiectasia, show signs of accelerated cardiovascular ageing, such as atherosclerosis, adverse myocardial remodelling, and overt CVD or CBVD at a young age.^11,12^ However, whether these conditions mirror physiologic ageing still requires clarification.

Telomere attrition is deeply intertwined with genome instability. Telomeres are nucleoproteic structures located at the extremities of chromosomes to counteract erosion of DNA occurring at each cell division.^13^ In germinal cells, telomere length is maintained by the nuclear telomerase machinery, whose function is adding telomeric sequences to chromosomes after each cell division.^14,15^ In human somatic cells, telomerase is usually inactive, and telomeres’ length is progressively reduced after each cell cycle. When telomeres’ length reaches a critical threshold, DNA repair mechanisms are activated and cells enter replicative senescence or apoptosis.^16^ Shortened telomeres in leucocytes have been observed in heart failure (HF), coronary artery disease (CAD), carotid atherosclerosis, and aortic stenosis,^17–20^ and a large Mendelian randomization study associated single-nucleotide polymorphisms (SNPs) in two components of the telomerase complex, namely *OBFC1* and *TERT*, to shorten telomeres and risk of ischaemic heart disease.^21^ In particular, *TERT* mutations and telomere shortening were associated with clonal haematopoiesis of indeterminate potential (CHIP), an emerging cardiovascular risk factor among the ageing population.^22^

### 2.2 Epigenetic dysregulation

Molecular mechanisms governing gene expression are collectively named ‘epigenetic’ and can be classified into two main categories: (i) mechanisms regulating gene transcription and (ii) mechanisms regulating messenger RNA (mRNA) translation. In turn, gene transcription is regulated by (i) molecular modifications of DNA and (ii) chromatin remodeling.^23^ The main DNA covalent modification is methylation of cytosine and adenine residues, with 80% of methylation sites occurring within a cytosine–guanine dinucleotide (CpG).^24,25^ The functional effect of DNA methylation varies according to genomic location and the cell type.^26^ Ageing is associated to specific methylation patterns, which constitute an epigenetic signature of ageing.^27^ Similarly, epigenetic signatures were associated to the risk of incident CVD.^28–30^ Since methylation patterns are inherited in around 9–19% of the total methylation CpG sites,^31^ the causal role of specific methylation patterns in CVD was demonstrated by Mendelian randomization studies.^31^

Chromatin remodelling is a dynamic process, regulating the access of transcription factors to specific genomic regions. Covalent modifications of histone proteins, such as acetylation, methylation, phosphorylation, carbonylation, and ADP-ribosylation, change the affinity of histones to DNA, thus promoting their condensation or disassociation.^32^ Furthermore, histone–DNA complexes can be actively formed, removed, or exchanged by ATP-dependent remodelling complexes.^33^ Ageing is associated to chromatin remodelling consisting of a generalized reduction of heterochromatin; at the same time, heterochromatin accumulates in specific genomic regions, resulting in an increased heterogeneity of chromatin condensation.^34^ These changes result in increased genomic instability, loss of silencing, and increased transcription of retrotransposons.^35,36^ In particular, modifications of histone protein 3 (H3) have been identified as key players by animal and human studies in myocardial hypertrophy, non-ischaemic dilated cardiomyopathy, and carotid atherosclerosis.^37–39^

Non-coding RNAs (ncRNAs) are RNA sequences of various lengths, not translated into proteins, which exert an ancillary function in protein synthesis. Regulatory ncRNAs can be classified in micro-RNAs (miRNAs, <200 nt), long ncRNA (lncRNAs, >200 nt), and circular RNAs (circRNAs).^40^ miRNAs bind the 3′ untranscribed region (3′UTR) of mRNA preventing the assembly of the ribosomal machinery and, therefore, inhibiting its translation. lncRNAs and circRNAs have, instead, a more complex range of functions. The expression of ncRNAs changes over a lifetime in a tissue-specific manner,^41–43^ and the expression of some ncRNAs was associated with lifespan in animals and humans.^41,44^ Some of these ncRNAs, such as the miRNA *miR-21* and the lncRNA H19, were also associated to CVD and CBVD.^40,44,45^

Since epigenetic mechanisms can be manipulated by pharmacological agents,^46,47^ lifestyle interventions,^48^ or diet,^49^ they represent a promising target for ageing-oriented treatments. However, epigenetic mechanisms show low specificity and, therefore, their manipulation carries a non-negligible risk for off-target effects.

### 2.3 Imbalance in energy metabolism

Ageing is characterized by a progressive imbalance in energy metabolism, both at cellular, organ, and organism levels. At the cellular level, this imbalance consists in a progressive loss of mitochondrial efficiency, resulting in an increased production of reactive oxygen species (ROS) and destabilization of the respiratory chain.^50^ Altogether these mechanisms predispose to genomic instability, cell death, and inflammation.^51^ ROS are highly reactive oxygen radicals, generated by the cellular aerobic metabolism, which can induce oxidation and peroxidation of other biological molecules, thereby compromising their function.^50^ To counteract oxidative stress, cells possess endogenous ROS scavenging systems, which neutralize free radicals; thus, low levels of endogenous antioxidants are associated with accelerated ageing.^52–54^ Endothelial dysfunction, the failure of the endothelium to induce vasodilation in response to hypoxia and haemodynamic stress, is a key early feature of vascular ageing and recognizes oxidative stress among its causes.^55,56^ Indeed, the most relevant molecular mediator of endothelial function is nitric oxide (NO), a soluble radical produced by the endothelial enzyme NO synthase (eNOS),^57^ whose function declines with age as a consequence of ROS accumulation, protein catabolism by-products, and increased arginases activity.^58–60^ Furthermore, most of the acute CVD and CBVD recognize ischaemia/reperfusion (I/R) injury as a core pathogenetic event, with the reperfusion phase characterized by a massive production of ROS, sustaining and propagating tissue damage.^61^ Finally, energetic derangements are a hallmark of cardiomyocyte dysfunction in HF, especially in HF with preserved ejection fraction (HFpEF), which is more prevalent with advanced age.^62^

At the organism level, energy metabolism imbalance consists in the dysregulation of nutrient-sensing mechanisms, constituted by the growth hormone (GH), insulin, glucagon, insulin-like growth factors (IGFs), glucagon-like peptides (GLPs), their membrane receptors, and downstream intracellular mechanisms.^9,63^ During childhood and adolescence, the nutrient-sensing system has an anabolic profile, characterized by the overexpression of GH, IGF1, and insulin, to promote the physiologic body accrual; however, the persistence of this anabolic profile during adulthood is associated with increased morbidity and reduced lifespan, whereas its inhibition increases lifespan and healthspan in different animal models.^64–66^ In humans, dysregulated nutrient-sensing are recapitulated by the metabolic syndrome, a combination of visceral obesity, insulin resistance, atherogenic dyslipidaemia, and cardiovascular disorders.^67^ Metabolic syndrome is characterized by accelerated epigenetic ageing and chronic low-grade inflammation, thus yielding a condition of accelerated ageing.^68,69^ Interestingly, ageing determines changes in body composition, with an increase in visceral fat mass and a reduction of muscle mass; this condition, also known as ‘sarcopenic obesity’ predisposes to metabolic syndrome and to an unfavourable energy metabolism.^70^

### 2.4 Impaired proteostasis and autophagy

Cells often generate defective proteins because of errors in genetic information, protein translation, and post-translational modification. Protein folding is a crucial process for protein assembly and functionality, and chaperone proteins oversee this process.^9^ Under physiological conditions, defective proteins are detected by the quality control mechanism and degraded by proteasome machinery or by autophagy.^71,72^ Excessive generation of defective proteins or their reduced degradation leads to their accumulation as intracellular or extracellular aggregates, causing cellular dysfunction and ageing.^73^ Consistently, in animal models, manipulation of protein homeostasis, by inducing chaperones activity, proteasome efficiency, or autophagy, results in prolonged lifespan.^74–76^ In the cardiovascular system, the paradigmatic example of a protein misfolding disease is represented by transthyretin (TTR) amyloidosis, a systemic disease with myocardial involvement, caused by deposition of misfolded TTR oligomers in the extracellular compartment, where they form amyloid plaques.^77^ Interestingly, only a minority of patients has an amyloidogenic mutation of the TTR gene; these patients are characterized by an early onset of symptoms and a different clinical presentation.^78^ On the other side, most of patients present no pathologic variant of the TTR gene and have a later onset of the disease, suggesting that the pathogenetic event occurs at a post-translational level and that ageing facilitates this pathologic process.

Impaired intracellular protein homeostasis has been associated to CVD, in particular MI, systemic hypertension, and HF.^79^ Likely, impaired proteostasis is a consequence of other pathologic features, such as disturbed energy metabolism or epigenetic modifications; however, it further impairs the overall cellular function, thus contributing to damage maintenance and propagation.^80^

Experiments in animal models demonstrated that rapamycin, inhibitor of the mammalian/mechanistic target of rapamycin (mTOR), and the polyamine compound spermidine promote autophagy. These compounds extend lifespan of experimental animals and exert beneficial effects on myocardial ischaemia and adverse myocardial remodelling.^81–83^ However, the observed improvement of proteostasis is probably only one of the cardio-protective mechanisms exerted by these compounds, since they also display pleiotropic biological effects including promotion of mitochondrial biogenesis, inhibition of the nutrient-sensing systems, and enhancement of NO production.^83,84^ These notwithstanding, promising results from pre-clinical studies with rapamycin were not confirmed in clinical trials,^85^ whereas the efficacy of spermidine has not been tested in clinical trials. Furthermore, the potential use of rapamycin in humans is limited by its anti-proliferative and immune-suppressant effects.^86^

### 2.5 Cellular senescence and stem cell exhaustion

Endogenous or exogenous stressors inducing a critical damage to proliferating cells lead to replicative arrest, to allow repair and prevent propagation of damaged/defective cells. If the repair mechanisms fail, the cell cycle cannot be restored and the cell enters senescence.^87^ The senescence-associated secretory profile (SASP) is characterized by growth factors, pro-inflammatory cytokines and chemokines, extracellular matrix proteases, and ROS. SASP maintains the senescence status in an autocrine manner, induces senescence in surrounding cells, and activates an immune response to clear out senescent cells.^88^ With ageing, cells are more prone to enter senescence, because of telomere attrition and accumulation of molecular damages; at the same time, with ageing, the removal of senescent cells becomes less and less efficient, resulting in their progressive accumulation within the tissues.^89^ Accumulation of senescent cells exerts a negative effect in adult life, as demonstrated by the increased health- and lifespan in murine models of spontaneous ageing, achieved by genetic or pharmacological elimination of senescent cells.^90^ Consistently, transplantation of senescent cells into young recipients reduces their lifespan.^90^ Senolytics are a class of drugs selectively removing senescent cells by inducing apoptosis, and two senolytic agents have a potential therapeutic use.^91^ The tyrosine kinase inhibitor dasatinib has mild senolytic effects in pre-adipocytes by inhibiting senescence-promoting proteins like p21 and B-cell lymphoma extra-large (Bcl-xL). However, the combination of dasatinib with the natural derivate antioxidant quercetin removes senescent endothelial cells *in vitro* and improves cardiac and endothelial function in naturally aged mice.^92^ Navitoclax, a selective inhibitor of the anti-apoptotic Bcl protein family, has senolytic activity in endothelial cells *in vitro*,^93^ prevents adverse myocardial remodelling, and reduces atherosclerotic burden in murine models of atherosclerosis and MI, respectively.^94,95^ Despite promising pre-clinical results, the efficacy of senolytics has not yet been tested in humans. However, two recent phase 1 trials demonstrated the safety and tolerability of dasatinib–quercetin and foselutoclax in patients with mild cognitive impairment and diabetic retinopathy, respectively.^96,97^

Since the pool of stem cells in each tissue is limited, senescence accounts also for the progressive depletion of stem cells and thus the reduction in tissue regenerating potential. Although myocardial stem cells have been identified, their role in heart diseases is debated, as adult myocardium has a very limited regenerative capacity.^98,99^ On the other hand, endothelial progenitor cells (EPCs) are well characterized, and they contribute to angiogenesis and vascular repair.^100^ EPCs are bone marrow–derived mesenchymal cells, and their circulating number correlates with cardiovascular risk and endothelial function.^101^ Despite these experimental findings, the potential for EPCs as therapeutic target in CVD was not confirmed in large clinical trials, which did not report convincing benefits for stem cell therapy in patients with MI or peripheral artery disease.^102,103^

### 2.6 Altered inter-cellular communication

The above-described ageing-associated cellular changes compromise inter-cellular communication. As a result, geropromoting factors are released in the extracellular milieu and in the blood stream, propagating an aged phenotype in the neighbouring and distant tissues. For instance, a single transfusion of blood from naturally aged mice can induce ageing features in young mice.^104^ Most of the geropromoting circulating factors are secreted in the context of SASP and, therefore, could be neutralized by senolytic treatments.^105^ Of special relevance for the cardiovascular system is the disrupted communication between the cellular component and the extracellular matrix (ECM). Ageing ECM is characterized by a progressive decline in biomechanical properties due to collagen fragmentation, oxidation, and glycation. Furthermore, ECM is affected by a progressive increase of enzymatic and non-enzymatic covalent bonds between molecules and by a progressive reduction of the elastin-to-collagen ratio. Finally, misfolded or pathologic proteins may infiltrate the ECM.^106^ These changes are primarily due to functional decay of fibroblasts, infiltration of immune cells, and circulating geropromoting factors.^107^ ECM remodelling has a secondary effect on surrounding cells, such as cardiomyocytes and endothelial cells, through mechano-transduction systems which result in increased cell senescence, impaired autophagy, and progressive mitochondrial dysfunction.^108^ In the cardiovascular system, this vicious circle translates into stiffening of the arterial and left ventricular wall, two main features of cardiovascular ageing.^109,110^ Matrix metalloproteinases (MMPs) play a pivotal role in ageing-related ECM remodeling,^111^ and therefore the potential benefits of MMP inhibitors in patients with CVD were tested in small trials, which retrieved promising, though inconclusive, results.^112,113^

### 2.7 Inflammation

Ageing is characterized by a dysregulation of the immune system triggering a persistent state of low-grade sterile inflammation.^114^ This condition, termed inflamm-ageing, is characterized by elevated levels of blood inflammatory markers and is recognized as risk a factor for cardiovascular diseases.^115^ A variety of potential mechanisms contribute to inflamm-ageing, including genetic susceptibility, central obesity, increased gut permeability, changes to microbiota composition, cellular senescence, Nod-like receptor protein 3 (NLRP3) inflammasome activation, oxidative stress, immune cell dysregulation, and chronic infections.^116,117^ Immunosenescence encompasses age-related disorders affecting both innate and the adaptive immune responses. Components of innate immunity show reduced efficacy of microbial killing and increased secretion of pro-inflammatory cytokines, extracellular matrix proteases, neutrophil extracellular traps, and reactive oxygen species,^118,119^ while adaptive immune system is characterized by a pro-inflammatory phenotype and increased trend towards autoimmunity.^120^ Immunosenescence leads to a reduced sensitivity to immunogenic stimuli and inadequate clearance of senescent cells, thus worsening the systemic exposure to pro-inflammatory SASP.^116^ NLRP3 inflammasome mediates the production/activation of the pro-inflammatory cytokines of the interleukin 1 (IL1) superfamily. The NLRP3 inflammasome is activated by unspecific danger-associated molecular patterns (DAMPs) like adenosine triphosphate, uric acid, and cholesterol crystals.^121^ Impairment of mitophagy and mitochondrial dysfunction contributes to inflammasome activation by inducing caspase-1, the enzyme cleaving pre-IL1 before it is secreted.^122^ In turn, caspase-1 inhibits mitophagy, originating a vicious cycle.^123^

The NLRP3 inflammasome plays a pivotal role in cardiovascular ageing. Indeed, antagonization of the NLRP3 inflammasome extends lifespan in naturally aged mice,^124^ while antagonization of IL1α and IL1β exerts a protective effect towards acute brain ischaemia and blunts arterial thrombosis in mice.^125–127^ This pre-clinical evidence is partially recapitulated by the results of the CANTOS trial, which demonstrated a reduction of major cardiovascular events in patients with very-high risk and chronic low-grade inflammation receiving the anti-IL1β antibody canakinumab.^128^ Beyond IL1, several pro-inflammatory cytokines demonstrated a geropromoting effect, including IL6 and tumour necrosis factor α (TNFα),^129^ although the net effect of their antagonization is not yet totally clear. For instance, antagonization of TNFα has a protective effect against acute brain ischaemia in naturally aged mice, restoring the phenotype of young mice;^130^ however, clinical trials in patients with heart failure demonstrated no benefit, with a potential increased risk of hospitalization for acute heart failure.^131,132^

### 2.8 Dysbiosis

The composition of gut microbiome depends on several factors like genetics, diet, lifestyle, and environmental conditions, making the microbiome unique for each individual.^133^ Age affects the microbiome composition; indeed, centenarians show a shift in microbiome composition, with a reduction in species typical of young subjects, like *Bacteroides* spp., and increases in alternative taxa like *Akkermansia* spp.^134^ However, persistence of *Bacteroides* spp. dominance or a low prevalence of alternative species with older age predicts decreased survival in a 4-year follow-up.^135^ This evidence suggests that gut microbiota adapts to the ageing host and that the reciprocal adaptation of host and symbionts promotes healthy ageing. The decline in beneficial bacteria like *Firmicutes* spp. is coupled with an increase in pro-inflammatory facultative anaerobes, releasing pro-inflammatory components such as lipopolysaccharide (LPS), which is eventually absorbed into the bloodstream contributing to chronic low-grade inflammation.^136^ With advanced age, this process is facilitated by the decay in colonic barrier function,^137^ suggesting that ageing is characterized by a higher susceptibility to changes in microbiome composition.

Beyond systemic inflammation, the microbiome influences cardiovascular function by releasing active metabolites. Circulating levels of trimethylamine N-oxide (TMAO), trimethyllysine, and aromatic derivates of amino acids, such as phenylacetylglutamine (PAG), have been associated with increased risk of major cardiovascular events.^138–140^ The molecular mechanisms linking these metabolites to CVD are still unclear, though accumulating evidence indicates a pro-thrombotic effect of TMAO.^141,142^ Furthermore, the predictivity of TMAO towards cardiovascular risk, over and beyond established risk factors, was confirmed by large clinical studies, and therefore, TMAO has been proposed as a potential predictive biomarker of CVD.^143^

The possibility to favourably manipulate microbiome composition by probiotics and prebiotics was explored in many studies; however, they usually included a low number of patients and suffered from a poor standardization of the intervention.^144^ Alternatively, microbiome composition can be modified by diet: for instance, TMAO is produced by the metabolism of choline and carnitine, whose content is more abundant in Western diet and relatively low in Mediterranean and plant-based diets.^145^

## 3. Diagnostic and therapeutic implications

### 3.1 Telomeres and telomerases

Although the causal role of telomere attrition in determining CVD and CBVD is still debated, leucocytes’ telomere length has been proposed as a tool to assess biological age,^146^ but its clinical implementation is hindered by a lack of standardization.^147^ Pharmacological telomerase activators were explored as potential ageing-oriented treatments. Since telomerase re-activation in somatic cells is associated with the acquisition of a malignant phenotype, cancer-related risk is the main conceptual limitation to the use of telomerase activators.^14^ However, mice with hyper-long telomeres do not demonstrate any increased risk of developing cancer, rather a risk reduction, alongside a better lipid and glucose metabolic profile.^148^ Pre-clinical evidence suggests that resveratrol, a natural derivate antioxidant, and statins may induce telomerase activity, by interacting with telomerase reverse transcriptase (TERT).^149,150^ The specific TERT activator AGS-499 increases extranuclear concentration of TERT and improves endothelial-mediated vasodilation in coronary arterioles of patients with CAD. However, this effect is likely secondary to the antioxidant effect of extranuclear TERT.^151^ Instead, the natural derivative compound TA-65® is an activator of the *Terc* gene, coding for the telomerase RNA component (TERC), which constitutes the template RNA sequence for TERT.^152^ TA-65 was shown to reduce inflammation after MI in a randomized clinical trial.^153^ Finally, several randomized clinical trials demonstrated that physical activity, in particular endurance activity, promotes telomerase activity by inducing TERT.^154,155^

### 3.2 Epigenetic clocks

Based on the association between methylation patterns and ageing, several machine learning algorithms, collectively named ‘epigenetic clocks’, have been proposed to estimate the age of a DNA source.^156^ Consistently, any discrepancy between epigenetic and chronological age of an organism could be regarded as an acceleration or deceleration of ageing, caused by pathologic conditions or environmental exposures.^157,158^ However, recent evidence suggests that epigenetic clocks trained on sufficiently large datasets would not show any significant departure from chronological age.^159^ Epigenetic age is an independent predictor of CVD, though limited by a small size effect.^160^ Furthermore, accelerated epigenetic ageing is associated with cardiovascular risk factors, such as overweight, insulin resistance, and hypertension, as well as pre-clinical atherosclerosis markers, such as carotid intima-media thickness and arterial stiffness.^160–163^ More potential blood-derived biomarkers of cardiovascular ageing are reported in *Table 1*.

**Table 1:** Potential molecular biomarkers of cardiovascular ageing

Name	Hallmarks of ageing	Evidence	Limitations	References
Leucocyte telomeres length	Telomere attrition	Predicts all-cause mortality and cardiovascular mortality Association with CAD, HF, atherosclerosis, and aortic stenosis in cross-sectional studies	Lack of method standardization	^17–19,146^
Epigenetic clocks	Epigenetic modifications	Predict cardiovascular mortality, MI, HF, and PAD Correlate with CV risk scores	Small effect size Size of training data sets	^160–163^
Sirtuins (Sirt1, Sirt2, Sirt6)	Epigenetic modifications Oxidative stress Cell senescence Inflammation	Association with lifespan in Mendelian randomization studies Sirt1 predicts microvascular dysfunction Sirt2 predicts CAVS Sirt6 predicts mortality in patients with AIS	Lack of data in general population Conflicting results in epidemiological studies	^164–173^
Monocyte chemoattractant protein 1 (MCP-1)	Intercellular communication Inflammation	Association with chronological age Predicts risk of stroke in Mendelian randomization studies Predicts CV mortality	Lack of specific studies on CV ageing	^174–176^
Vascular cell adhesion molecule 1 (VCAM-1)	Intercellular communication Inflammation	Association with chronological age Association with worsening myocardial function	No association with CV risk	^174,177–180^
Interleukin 6 (IL6)	Inflammation	Association with chronological age Association with arterial stiffness Predicts CV risk in mendelian randomization studies	Lack of specific studies on CV ageing	^174,181,182^
Trimethylamine N-oxide (TMAO)	Dysbiosis	Association with chronological age Association with arterial thrombosis Predicts CV risk	Lack of specifictity for the cardiovascular system	^139,141–143,183,184^

Blood-derived biomarkers are easily accessible biological samples and may represent a measure of one or more hallmarks of ageing.

AIS, acute ischaemic stroke; CAD, coronary artery disease; CAVS, calcific aortic valve stenosis; CV, cardiovascular; HF, heart failure; MI, myocardial infarction; PAD, peripheral artery disease.

### 3.3 Sirtuins

Sirtuins (Sirt) are a family of seven nicotinamide adenine dinucleotide (NAD^+^)-dependent enzymes, highly conserved throughout evolution and showing a high degree of homology.^185^ The main biochemical function of Sirtuins is deacetylation of lysine residues in histone and non-histone proteins, and they are therefore classified as class III histone deacetylases.^46^ According to their intracellular localization, Sirtuins can be classified as nuclear (Sirt1, Sirt6, and Sirt7), cytoplasmic (Sirt2), or mitochondrial (Sirt3, Sirt4, Sirt5).^186^ At a genomic level, Sirtuins promote the function of DNA repair machinery and the elongation of telomeres,^187^ but they are also involved in energy homeostasis, glucose metabolism, cellular senescence, and systemic inflammation.^188,189^ Thus, Sirtuins regulate multiple hallmarks of ageing, and, indeed, their function declines with ageing.^190^ Sirtuins 1 and 6 have been demonstrated to promote lifespan and healthspan in animal models,^164,165^ and the association between Sirt6 and human lifespan was confirmed in genome-wide association studies (GWAS).^166^ Interestingly, mitochondrial Sirtuins seem to have an opposite effect on lifespan: Sirt3 deficiency increases lifespan in mice, and single-nucleotide polymorphisms (SNPs) inducing loss of function in *Sirt5* gene are associated with prolonged lifespan in healthy humans.^167,191^ SNPs in the *Sirt3* gene are also associated with lifespan, although the functional effect of these variants is unknown.^192,193^ The relationship between Sirtuins and CVD/CBVD is more complex: intracellular overexpression of Sirt1 is protective towards CAD and carotid atherosclerosis,^168,169,194^ while circulating levels of Sirt1 show an opposite trend.^169^ Sirt6 protects against acute brain ischaemia,^170^ although observational studies reported a U-shaped relationship between circulating Sirt6 and mortality in patients with acute ischaemic stroke.^195^ Conversely, Sirt5 promotes acute arterial thrombosis and exerts a detrimental effect in acute brain ischaemia.^196,197^ Finally, Sirt3 is protective in acute myocardial ischaemia and myocardial hypertrophy.^198,199^ Since Sirtuins’ activity strictly depends on NAD^+^ whose availability declines with ageing, NAD^+^ supplementation is the most obvious strategy to enhance their activity. Animal studies reported that the administration of NAD^+^ or its precursors, such as nicotinamide (NAM), nicotinamide mononucleotide (NMN), or nicotinamide riboside, can improve healthspan, cardiac function in HFpEF, cognitive performance in chronic cerebral hypoperfusion, and blood–brain barrier integrity.^200–203^ Recent trials showed that oral supplementation of NMN improves insulin sensitivity in pre-diabetic women,^204^ while NAM reduced the risk of developing acute kidney injury in critically ill patients.^205^ However, human data about the effect of NAM or NMN on CVD are not yet available. Overall, an observational study revealed that nutritional assumption of NAD^+^ precursors is associated with a reduced risk of cardiovascular death.^206^ Natural-derived flavonoids, such as resveratrol and quercetin, have several biological effects, including induction of Sirtuins’ transcription.^207^ Furthermore, resveratrol inhibits the degradation of NAD+ by phosphodiesterases, promotes the binding of Sirt1 to its nuclear activator lamin A, and promotes the expression of eNOS in a both Sirtuin-dependent and independent manners.^208–210^ By inducing eNOS, resveratrol improves endothelial function in animal models of systemic hypertension and diabetes mellitus^211,212^; this effect was also confirmed in a small trial in obese/overweight subjects.^213^ However, another small trial in a similar population of patients did not confirm any improvement in blood glucose profile, body composition, or blood pressure.^214^ The lack of large clinical studies prevents us from drawing conclusions about effects of resveratrol on glucose metabolism or vascular function. The main limitations of nutritional supplements such as NMN and resveratrol are poor bioavailability, due to limited absorption and high extracellular metabolism, and potential interaction with other medications. Furthermore, they are unspecific activators of Sirtuins: as described above, different Sirtuins may have conflicting effects on ageing and CVD/CBVD, resulting effects that are to date unpredictable. To overcome this limitation, non-flavonoid activators of Sirt1 are under investigation, but they have not yet been tested in clinical trials.^215^

### 3.4 Antioxidants

Antioxidants can be classified in two broad categories: (i) endogenous, such as glutathione or coenzyme Q10, or (ii) exogenous, such as flavonoids and vitamin E.^216^ The efficacy of antioxidants in preventing overt CVD has been tested in several trials, with often conflicting results.^217–220^ The unsuccessful implementation of antioxidants could be attributed to a lack of specificity: indeed, their mechanism of action is based on scavenging ROS or reducing their production. However, ROS are constitutively produced by cellular aerobic metabolism, and they exert relevant physiologic functions, like promoting cell survival and differentiation.^221,222^ Therefore, ROS scavenging may result in a compensatory overproduction, without a relevant effect on the overall redox balance. As proposed by Sies and Jones,^221^ controlling specific ROS-mediated signalling pathways may offer a new perspective in precision medicine. For instance, the adaptor protein p66^Shc^ represents the molecular link between oxidative stress and programmed cell death. In response to different cellular stressors, p66^Shc^ is activated and relocates from the cytosol to the mitochondria, where it promotes the generation of ROS and the activation of the mitochondrial apoptosis pathway.^223^ Genetic deletion of p66^Shc^ promotes cellular resistance to ROS-induced apoptosis *in vitro*, prolongs lifespan and healthspan in mice, and prevents age-dependent endothelial dysfunction,^224,225^ suggesting that p66^Shc^ regulates cardiovascular ageing by modulating the response to oxidative stress. Interestingly, inhibition of p66^Shc^ has a protective effect in brain acute ischaemia,^226,227^ but has a neutral or even detrimental effect on MI.^228,229^ A similar role, though with an opposite functional effect, was attributed to the transcription factor JunD, which promotes the transcription of endogenous antioxidants and hypoxia-responsive proteins.^230^ Genetic deletion of JunD in mice leads to reduced lifespan, likely mediated by overexpression in IGF-1.^231^ Furthermore, spontaneous ageing is associated with a reduced expression of JunD, and JunD knock-out mice express an accelerated decay in endothelial function.^232^ Like p66^Shc^, also JunD seems to have an organ-specific effect in I/R damage. Indeed, whereas JunD overexpression worsens MI by downregulating Sirt3,^233^ it has a protective effect in acute brain ischaemia. Interestingly, JunD silencing determines a pro-inflammatory status after acute brain ischaemia and the detrimental effect of JunD deficiency can be counteracted by anti-inflammatory agents.^234^ This observation suggests that JunD could be a molecular link between oxidative stress and inflammation. Despite these interesting pre-clinical results, pharmacological agents regulating the activity of p66^Shc^ or JunD have not been tested yet in clinical trials.

### 3.5 Nutrition and physical activity

Caloric restriction (CR), defined as a reduction in caloric intake of 20–40% compared to a normal *ad libitum* diet without compromising essential nutrient intake, is the most effective non-pharmacological intervention to increase lifespan in animals, including non-human primates.^235,236^ Mechanistically, CR acts on multiple biochemical pathways associated with ageing: (i) it inhibits the insulin/IGF1 pathway, (ii) modulates the intracellular energy regulators, (iii) promotes the expression of Sirt1, and (iv) induces autophagy.^237^ The implementation of CR in humans is limited by a reduced compliance, especially over extended periods of time; however, positive effects on immune system and inflammation were demonstrated in healthy humans after 2 years of moderate CR.^238^ Alternative dietary regimens mimicking the molecular effects of CR have been investigated: intermittent fasting (IF) collects a wide range of dietary regimens, characterized by usual *ad libitum* feeding, interrupted by fasting for 12–24 h at regular intervals.^239^ Although intermittent fasting increases lifespan in *Drosophila*,^240^ this could not be confirmed in mammals. Furthermore, a recent meta-analysis of 18 human trials demonstrated essentially a neutral effect of IF on cardiometabolic risk, beyond weight loss.^239^ Ketogenic diets (KD) are a group of dietary regimens characterized by a restriction of carbohydrate intake (<50% of daily caloric intake) in the context of a variable restriction of caloric intake,^241^ promoting a shift of energy metabolism from catabolism of carbohydrates to triglycerides, with the formation of ketone bodies.^242^ Although administration of β-hydroxybutyrate in drinking water extends lifespan in mice,^243^ no significant cardiovascular effect of KD was observed in clinical trials, beyond body weight loss.^244,245^ Alternatively, molecular mechanisms elicited by CR can be mimicked by pharmacological agents acting on oxidative stress, autophagy, intracellular glucose bioavailability or intracellular nutrient sensing.^237^ For instance, the anti-ageing properties of metformin, an insulin-sensitizing biguanide derivate, have been observed in pre-clinical investigations.^246^ However, metformin has not been proven to reduce the incidence of CVD in subjects with impaired glucose tolerance, in spite of effective diabetes prevention and reduction of systemic inflammation.^247,248^ In this context, novel antidiabetic drugs, namely, GLP-1 receptor agonists and sodium glucose co-transporter 2 (SGLT2) inhibitors, can be included among modulators of the nutrient-sensing mechanisms and their success in reducing cardiovascular risk over and beyond their antidiabetic effect may be an ageing-oriented mechanism of action.^249,250^

The benefits of physical activity (PA) for a healthy ageing are well established, in particular for the cardiovascular system.^251,252^ Physical activity is associated with reduced all-cause and cardiovascular mortality among old people; this effect is maximized with moderate-to-intense PA, whereas additional increase in intensity does not bring additional benefit.^253^ On a molecular level, regular PA acts on several hallmarks of ageing, as it (i) improves both insulin-dependent and insulin-independent glucose uptake, (ii) promotes mitochondrial biogenesis, (iii) improves proteostasis, and (iv) counteracts chronic low-grade inflammation.^171,254,255^ Importantly, PA is not associated *per se* with specific health risks even for moderate-to-high intensity in old people.^251^ Although engagement in competitive sports activity is associated with an increased risk of atrial fibrillation, non-competitive moderate-to-vigorous PA actually prevents the onset of this common heart rhythm disturbance.^256,257^

### 3.6 Anti-inflammatory drugs

The possibility to target systemic inflammation to prevent or treat CVD and CBVD is nowadays a concrete option. Due to moderate efficacy in preventing cardiovascular events and a slight risk in severe infections risk, the anti-IL1β antibody canakinumab has never entered clinical practice for this indication.^128^ Indeed, the beneficial effects of immunomodulatory drugs for prevention of CVD and CBVD are often burdened by the increased risk of severe infections, especially patients above 80 years-old.^129^ Low-dose colchicine on the other hand, seems to have addressed this burden and accordingly was recently approved for this indication by the Food and Drug Administration.^258^ The clinical use of colchicine is limited by its potential nephrotoxicity, in particular among the elderly who frequently present chronic kidney disease. To overcome this limitation, the direct inhibition of IL6 by ziltivekimab is currently under investigation in a phase IIb clinical trial, after the positive results of the phase IIa. Compared to IL6 receptor inhibitors, ziltivekimab is not expected to cause clinically relevant immunosuppression.^172,259^

## 4. Conclusions

**Figure 3:**
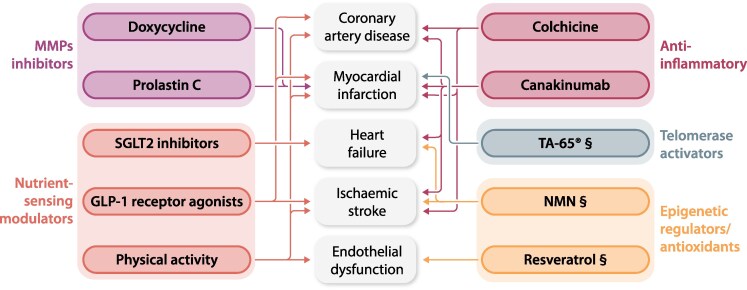
Pharmacological interventions addressing hallmarks of ageing with proven efficacy in clinical trials. Interventions are depicted as coloured ovals. Interventions addressing the same hallmark have the same colour. Arrows connect interventions (coloured ovals) to cardiovascular diseases or cardiovascular risk factors. GLP-1, glucagon-like peptide 1; NMN, nicotinamide mononucleotide; SGLT2, sodium glucose co-transporter 2. § means evidence is limited to small and/or conflicting trials.

The expanding knowledge about ageing provides us with a better understanding of this complex biological process with multifaceted effect on individual health. This notwithstanding, currently no intervention has been proven to reverse multi-system ageing in humans, underscoring the fact that this remains an important unmet clinical need. However, this knowledge can nowadays be acted upon to improve the management of age-related diseases, in particular CVD and CBVD, and the overall quality-of-life of an ageing population. As we have reviewed, the hallmarks of ageing can be addressed by pharmacological (*Figure 3*) and non-pharmacological interventions. With the significant exceptions of SGLT2 inhibitors, GLP1 receptor agonists, and colchicine, pharmacological interventions to counteract the hallmarks of ageing are still at an experimental stage, while nutritional supplements like NMN, resveratrol, or quercetin are easily available, but their effectiveness is only supported by small studies. On the other hand, non-pharmacological interventions like Mediterranean diet and moderate-intensity physical activity have proven their efficacy and safety with a robust level of evidence, also in advanced age. These actions represent the cornerstone for healthy ageing and should be recommended on a large scale. An important unmet clinical need is still represented by the assessment of biological age. Telomere length and epigenetic clocks have been proposed as measurements of biological ageing, but their accuracy and reproducibility are still uncertain. Probably, a single measurement is insufficient to cover the complexity of ageing, and a multidimensional assessment, integrating clinical and biochemical data, is needed.

## Authors’ contributions

G.G.C. and T.F.L. conceptualized the review and proofread the draft. S.M. and F.A.W. drafted the paper and the figures. All authors approved the final manuscript.

## Data Availability

No new data were generated or analysed in support of this research.
